# Community solutions to food apartheid: A spatial analysis of community food-growing spaces and neighborhood demographics in Philadelphia

**DOI:** 10.1016/j.socscimed.2022.115221

**Published:** 2022-08-05

**Authors:** Ashley B. Gripper, Rachel Nethery, Tori L. Cowger, Monica White, Ichiro Kawachi, Gary Adamkiewicz

**Affiliations:** a The Ubuntu Center on Racism, Global Movements, and Population Health Equity, Drexel Dornsife School of Public Health, Philadelphia, PA, United States; b Department of Environmental Health, Harvard T.H. Chan School of Public Health, Boston, MA, United States; c Department of Community Health and Prevention, Drexel Dornsife School of Public Health, Philadelphia, PA, United States; d Department of Environmental and Occupational Health, Drexel Dornsife School of Public Health, Philadelphia, PA, United States; e Department of Biostatistics, Harvard T.H. Chan School of Public Health, Boston, MA, United States; f Department of Social and Behavioral Sciences, Harvard T.H. Chan School of Public Health, Boston, MA, United States; g Department of Epidemiology, Harvard T.H. Chan School of Public Health, Boston, MA, United States; h FXB Center for Health and Human Rights, Harvard T.H. Chan School of Public Health, Boston, MA, United States; i Department of Community and Environmental Sociology, College of Agricultural and Life Sciences, University of Wisconsin-Madison, Madison, WI, United States

**Keywords:** Community gardens, Neighborhoods, Environmental racism, Structural racism, Environmental health, Urban agriculture, Spatial analysis, Philadelphia

## Abstract

Black and low-income neighborhoods tend to have higher concentrations of fast-food restaurants and low produce supply stores. Limited access to and consumption of nutrient-rich foods is associated with poor health outcomes. Given the realities of food access, many members within the Black communities grow food as a strategy of resistance to food apartheid, and for the healing and self-determination that agriculture offers.

In this paper, we unpack the history of Black people, agriculture, and land in the United States. In addition to our brief historical review, we conduct a descriptive epidemiologic study of community food-growing spaces, food access, and neighborhood racial composition in present day Philadelphia. We leverage one of the few existing datasets that systematically documents community food-growing locations throughout a major US city. By applying spatial regression techniques, we use conditional autoregressive models to determine if there are spatial associations between Black neighborhoods, poverty, food access, and urban agriculture in Philadelphia.

Fully adjusted spatial models showed significant associations between Black neighborhoods and urban agriculture (RR: 1.28, 95% CI = 1.03, 1.59) and poverty and urban agriculture (RR: 1.27, 95% CI = 1.1, 1.46). The association between low food access and the presence of urban agriculture was generally increased across neighborhoods with a higher proportion of Black residents.

These results show that Philadelphia neighborhoods with higher populations of Black people and neighborhoods with lower incomes, on average, tend to have more community gardens and urban farms. While the garden data is non-temporal and non-causal, one possible explanation for these findings, in alignment with what Philadelphia growers have claimed, is that urban agriculture may be a manifestation of collective agency and community resistance in Black and low-income communities, particularly in neighborhoods with low food access.

## 1. Introduction

Black and low-income neighborhoods tend to have higher concentrations of fast-food restaurants and low produce supply stores (Larson et al., 2009; Jeffery and French, 1998; Block et al., 2004; Berger et al., 2019). These stores and restaurants often have few food options that are healthy and nutritious. In Philadelphia, over “81 percent of food stores offer mostly unhealthy food choices.” (Neighborhood Food Retail, 2021) Limited access to and consumption of nutrient-rich foods is associated with poor health outcomes such as heart disease, diabetes (The top 10 leading causes, 2019; Zenk et al., 2005), depression, and anxiety (Fang et al., 2021).

Inadequate access to nutrient-rich, affordable, and culturally significant foods is a type of structural racism. According to Zinzi Bailey et Al., structural racism is the way that racism is “produced and reproduced by laws, rules, and practices, sanctioned and even implemented by various levels of government, and embedded in the economic system as well as in cultural and societal norms.” (Bailey et al., 2021; Bailey et al., 2017; Rothstein, 2017) Furthermore, Bailey and co-authors argue that “confronting racism, therefore, requires not only changing individual attitudes, but also transforming and dismantling the policies and institutions that undergird the U.S racial hierarchy.” (Bailey et al., 2021).

Given the realities of food access described above, many members within Black communities grow food as a strategy of resistance to food apartheid, and for the healing and self-determination that agriculture offers (Gripper, 2020). In contrast to the concept of food deserts, food apartheid refers to the racist structures, systems, and institutions that have led to an inadequate, inequitable, and unjust food environment (Brones, 2018) for Black Americans and other marginalized groups.

In cities across the United States, the presence of community food-growing spaces (gardens and farms) has increased (Here’s the dirt on park, 2021; Borowiak et al., 2018). Studies consistently show the social, cultural, and nutritional benefits of being a part of a community garden. Community gardening is linked to more fruit and vegetable consumption, lower rates of depression and anxiety, and better overall health outcomes (Draper and Freedmand, 2010; Girard et al., 2012; McCormack et al., 2010; Litt et al., 1021; Alaimo et al., 2016; George, 2013; Lampert et al., 2021; Gregis et al., 2021). Although, an increasing body of literature provides evidence that the leadership of food-growing spaces also matters (Golden, 2013; Bradley and Galt, 2014; White, 2010; McClintock, 2014). There may be a difference in the impact of urban, community food-growing depending on if a project is community-led or led by people from outside of the community (Golden, 2013; Bradley and Galt, 2014; McClintock, 2014).

Prior research on community gardens, race, and health is largely qualitative and comes primarily out of sociological, geography, political, and anthropological disciplines (Draper and Freedmand, 2010; Garcia et al., 2018). This research has added tremendous contributions to the literature on the impacts of urban agriculture, particularly on Black communities. While this body of literature includes much rigorous qualitative research, it lacks rigorous quantitative measurement and evaluation of the qualitative theories that have emerged (Kondo et al., 2018; Audate et al., 2019; Burt et al., 2021; Ohly et al., 2016).

Few peer-reviewed, published epidemiologic studies have assessed the association between Black neighborhoods, urban food-growing spaces, and socioeconomic variables (Audate et al., 2019; Burt et al., 2021). Even fewer studies have investigated the associations between urban food-growing spaces and the health of growers (Draper and Freedmand, 2010; Gregis et al., 2021; Audate et al., 2019). Of the studies that have assessed urban food-growing, neighborhood demographics, and health, most of them take place outside of the United States (Audate et al., 2019). Investigating the relationships between Black people, agriculture, and health could help clarify potential areas for political, economic, and policy support that leads to improvements in the overall health and wellbeing of this community.

Within a US context, one major obstacle to studying these associations at the population level is the limited documentation about locations and activities of community gardens. The quantitative studies that have examined the activities of community food-growing spaces rarely include analysis of the historical and sociopolitical contexts that have shaped these relationships. At present, rigorous quantitative analyses of the factors that shape community food-growing spaces are rare.

In this paper, we start by unpacking the history of Black people, agriculture, and land in the United States. This context is important to help our audience understand that Black people growing in cities is not a new phenomenon or trend, but something that has been done for practical, cultural, and spiritual reasons for many decades (Gripper, 2020). It will also help people understand that collective food-growing spaces are often forms of community care and constructive resistance to oppression and environmental racism (Gripper, 2020; White, 2018).

According to Robert Bullard, “environmental racism refers to any policy, practice, or directive that differentially affects or disadvantages individuals, groups, or communities based on race or color. It also includes exclusionary and restrictive practices that limit participation by people of color in decision-making boards, commissions, and regulatory bodies.” (Bullard, 1993) Generally, environmental racism refers to policies, practices, or actions that are directly related to the environment such as transportation routes, zoning ordinances, redlining, green space, and food access.

While much of the overall epidemiologic research highlights behavioral choices as the causes of poor health outcomes related to food, and more recent epidemiologic literature points to social determinants of health, we suggest that it is food apartheid and structural racism, manifesting as environmental racism, that lead to greater rates of diet-related illnesses (Odoms-Young and Bruce, 2018).

In addition to our historical review, we conduct a descriptive epidemiologic study of community food-growing spaces, low food access, and neighborhood demographics in present day Philadelphia. There has been a consistent and sustained presence of community gardening and urban farming in Philadelphia (Borowiak et al., 2018), however, the population health of Philly’s urban food-growing community has not been extensively documented. Although this study does not explicitly look at the impacts of urban food-growing on the health of Philly growers, we conduct an ecological analysis of variables that are consistently associated with mental and physical health – low food access, poverty, and neighborhood racial composition.

We leverage one of the few existing datasets that systematically documents all community food-growing locations throughout a major U. S. city. By applying spatial regression techniques, we use conditional autoregressive models to determine if there are spatial associations between Black neighborhoods, poverty, food access and urban agriculture in Philadelphia. Understanding the landscape of community food-growing spaces in the city will lend context to the role that these spaces have in shaping health outcomes, collective agency, and urban policies. In our conclusion, we offer recommendations on how to support urban agriculture and community food policies that may help disrupt and dismantle racism in Philadelphia’s food system.

## 2. Background – a brief history of structural racism experienced by black farmers

Black people have used agriculture to develop self-determined communities in the U.S. for over 150 years (Gripper, 2020; Daniel, 2013). In the decades immediately following the Civil War, Black people sought to acquire land to provide for their families, their communities, and to become independent of former enslavers and unjust conditions (Daniel, 2013; Tatah, 2017). However, they faced many obstacles in the quest to own and keep land, such as exploitative sharecropping and rental agreements. Denials of access to private credit often forced Black farmers to sell their land for far less than its value (Daniel, 2013). The USDA has admitted to engaging in racially discriminatory lending practices that impacted Black land access and ownership (Daniel, 2013; Tyler and Moore, 2013).

Despite the state-sanctioned efforts to thwart and exploit them described above, Black people acquired over 16 million acres of land; and in 1920, there were over one million Black farmers, even more if you include sharecroppers, who made up 14% of the farming population (Rosenberg and Stucki, 2019). Over the proceeding decades, white terrorism, Jim Crow laws, USDA discrimination, and increased industrialization in northern regions drove many Black people from the South to cities like Philadelphia, St. Louis, and Detroit (Great Migration, 2019; Tolnay, 2003). Those who were able to stay with their land and communities in the South faced incredible discrimination and racism through policies and procedures at the USDA (Rosenberg and Stucki, 2019). Black farmers were routinely and deliberately denied farm loans and crop insurance. When they fell behind on payments and yields because of predatory terms and a lack of necessary farming equipment, Black farmers were often forcefully removed from their land (One Million Black Families in, 2019).

Heirs’ property is another legal procedure that preyed on vulnerability and was used to steal Black land (Douglas, 2017). The USDA defines “Heirs’ property as land that has been passed down informally from generation-to-generation. In most cases, it involves landowners who died without a will. Heirs’ property is land owned ‘in common’ by all the heirs, regardless of whether they live on the land, pay taxes, or have ever set foot on the land.” This legal process allows developers and real estate agents to prey on families divided on how to proceed with land, sometimes convincing them into selling the entire property for less than its true value.

These measures stole so much Black land that from 1920 to 1997, the number of Black farmers declined by about 95% nationwide (Ficara and Williams, 2005). Since the early 1900s, Black farmers have suffered land theft of around 80–90%, while white landowners only experienced a 2% loss (Rosenberg and Stucki, 2019); and in 2017, there were only about 45,000 Black farmers in the U.S., making up just over 1% of the farming population (Daniel, 2013).

### 2.1. Community resistance to extraction and structural racism

As Black people migrated north because of Jim Crow, terrorism, and land theft in Southern states, they brought their heritage of growing food and stewarding land with them.

In cities across the United States, there are Black-led community food-growing projects that work towards food justice. Food justice occurs when communities have the rights and resources to grow, sell, and eat high quality foods that are culturally relevant, ethically produced, affordable, and nutritious (Alkon and Agyeman, 2011). Food justice is a movement that should be led by the people and communities who have been most deeply impacted by agribusiness, environmental racism, and food inequity (Alkon and Agyeman, 2011; White, 2011). We offer that this concept should also include the consideration of all Earth’s inhabitants – animals, plants, soil, microbes, and insects – and our interdependence.

Philadelphia’s Black and immigrant communities have a long and rich history of food justice and community gardening (Borowiak et al., 2018). City-wide vacant lot greening was encouraged and first recorded in 1897 (Speirs, 1898; Laskawy, 2010) and continued during and after World War II in the form of Victory Gardens (Laskawy, 2010). During these time periods, the City of Philadelphia offered infrastructural support to community members who wanted to practice self-reliance and grow their own foods on abandoned and unused land (Speirs, 1898; Laskawy, 2010; Nairn and Vitiello, 2009).

The era of deindustrialization in Philadelphia began in the late 1800s and continued well in to the 1970s and 80s (Nairn and Vitiello, 2009; Nairn, 2007; Birch and Wachter, 2008; Scranton, 1992; Park and Ciorici, 2013). A city that once hosted automobile and electric industries such as Ford and GE, soon found itself with fewer companies leading to fewer jobs for its residents (Birch and Wachter, 2008; Scranton, 1992). Post WWII, the population in Philadelphia decreased from about two million people to 400,000 (Birch and Wachter, 2008).

Between the 1950s and 1990s, several things happened in Philadelphia. There were waves of migration to Philadelphia by Black people from the South and Puerto Rican and Southeast Asian immigrants (Nairn, 2007; Park and Ciorici, 2013). At the same time, white home and landowners fled the city en masse and moved to surrounding suburban areas. The sharp decrease in population and loss of industry left thousands of vacant lots and abandoned homes in the city (Birch and Wachter, 2008). For five consecutive decades – from 1950 to late 1990s – Philadelphia experienced population decline. The 2000’s marked the first decade in which Philadelphia began to experience population growth again (Gyourko et al., 2005).

Philadelphia also began to experience another more permanent wave of urban growing. While crack and opioid epidemics devastated neighborhoods, many Black and immigrant communities reclaimed abandoned lots as spaces to grow food for themselves and their neighbors (Vitiello and Nairn, 2008). Throughout the 60s, 70s, and 80s, organizations such as Neighborhood Gardens Association and Penn State Extension were established and/or expanded their work to support Philadelphians who wanted to build gardens and growing spaces in their neighborhoods. Although a comprehensive recollection of Philadelphia’s community gardening history does not exist among academic publications, a more detailed history of community gardening in Philadelphia between the 1950’s and early 2000’s can be found in Vitiello and Nairn’s “2008 Harvest Report.” (Vitiello and Nairn, 2008).

For a time, Philadelphia city government was in support of these efforts since they help to beautify neighborhoods and curb the aesthetically unpleasing effects of extraction. During the 1990s, however, the City began to decrease its infrastructural and programmatic support for community-led agriculture projects (Laskawy, 2010), even though Philadelphia still had 31,000 vacant lots and 26,000 abandoned homes on record at the time (Birch and Wachter, 2008). The United States Congress also cut USDA support for urban agriculture programs (Vitiello and Nairn, 2008). This period coincided with several occurrences in Philadelphia: growth in population, a rise in the percentage of people with a bachelor’s degree, and an increase in the percentage of people without a high school diploma. From the 1990s–2000s, Philadelphia grew in the number of residents with college degrees and also grew in the amount of residents without high school diplomas (Gyourko et al., 2005). Essentially, Philadelphia’s decrease in infrastructural support of community-led agriculture projects happened around the same time as some of the city’s earliest waves of gentrification in working-class African-American neighborhoods (Changing Neighborhoods, 2016). Although we have been unable to locate records of precisely *why* city agencies began decreasing support of agriculture between the 1990s and 2000s, based on what else was happening in the city, it is reasonable to suggest that the decrease in support may have been influenced by the City’s desire for development that would attract highly educated new residents with higher incomes than the current population. Doing so would have helped to transition Philadelphia from continuous population decline into a consumer city (Gyourko et al., 2005).

Despite decreased support from city agencies, Black and immigrant communities in Philadelphia continued to reclaim vacant lots and return to their agricultural roots, practices, and foods (Laskawy, 2010; Nairn, 2007; Vitiello and Nairn, 2008). According to Vitiello and Nairn, at one time there were over two thousand greening projects throughout the city (Vitiello and Nairn, 2008) demonstrating the massive amounts of social capital and networks flowing in Philadelphia.

Today, Philadelphia now has over 418 community gardens, growing spaces, and urban farms (DiStasi et al., 2021). Transforming vacant lots into growing spaces beautifies blocks that have been neglected by the City’s agencies, officials, and private owners/developers, while providing so many other benefits to communities. In Philadelphia, collective food-growing projects facilitate healing and community building through access to nutrient-rich food, agricultural education, organizing, and social gatherings. The transformation of trash-ridden lots to gardens has been the fabric that strengthens so many Black neighborhoods in Philadelphia.

Unfortunately, many of the current community growing spaces in Philly are actively being threatened due to rapid development, gentrification, and displacement (Strauss, 2019; Briggs, 2021). For decades, the city experienced much neighborhood disinvestment – resulting in many abandoned buildings and about 40,000 vacant lots (Vacant Lot Program | Community Life Improvement Program, 2021). Growers in the city have collectively mobilized to turn some of these vacant lots into gardens and farms to feed and support their communities (Vitiello and Nairn, 2008; Strauss, 2019). Philadelphia still has thousands of vacant lots that could be transformed into spaces to offer affordable, nutritious, and culturally relevant food for its residents experiencing food apartheid.

## 3. Methods

### 3.1. Independent variables

We linked three spatially referenced data sources for Philadelphia: community garden and farm locations, neighborhood food retail access, and U.S. Census demographic data. For each census block group in Philadelphia, data on racial composition, mean property value, population density, and total population were obtained from the US Census Bureau’s 2016 American Community Survey (ACS). ACS data were downloaded through the National Historical Geographic Information System (Manson et al., 2021). We collected block group level measures of poverty and neighborhood food access from the 2019 report on Neighborhood Food Retail in Philadelphia (Neighborhood Food Retail, 2021). The sociodemographic and food access variables serve as independent variables in our analyses. There was a total of 1324 block groups in Philadelphia for which there was data on all independent variables. Block groups were excluded if there was no data available on independent variables (N = 12).

Independent variables include predominantly Black neighborhoods, poverty, and low food access assessed at the block group level. Black neighborhoods were measured as the percent of a block group identifying as Black. Poverty was defined as the percent of a block group living below 200% of the federal poverty limit. We adjusted analyses for mean property value and population density. These covariates were identified a priori and are commonly adjusted for in spatial analyses at the group level.

The Philadelphia Report on Neighborhood Food Retail has several measures for food access (Neighborhood Food Retail, 2021). These include a categorical variable measuring “no access,” “low access,” and “moderate/high access.” We are concerned that collapsing moderate and high food access into one category will miss nuances of the food environment such as quality of produce and quality of other foods. Some supermarkets and grocery stores sell mostly poor quality or moldy produce. Some grocery stores sell mostly fast-food that is low in nutrients. This way of accounting for food access might categorize a block group with poor quality supermarkets as “moderate/high food access”, when the quality and type of food should qualify the block group as “low food access.” These categories do not capture the information we are looking for.

For this reason, we chose “number of low-produce supply stores” (LPSS) as the low food access variable due to potential measurement error in the other food access variables, including the high produce supply store and categorical food access variables. The LPSS block group measure represented the number of low produce supply stores within walking distance per 1000 people. Walking distance was defined as “within ½ mile walking along the street.” (Neighborhood Food Retail, 2021) The Philadelphia Report on Neighborhood Food Retail defined low-produce supply stores as “stores that generally offer a low amount of health food choices and often have a higher amount of unhealthy food choices, like sugary beverages and sugary and salty snacks.” (Neighborhood Food Retail, 2021) More information on data collection methods can be found in the report on Neighborhood Food Retail through OpenDataPhilly.

### 3.2. Dependent variables

Point and polygon data of community gardens and urban farms were obtained from the Public Interest Law Center’s Garden Legal Justice Initiative dataset, which is managed by the Garden Data Collaborative (GDC) (Bouffard, 2017). GDC was formed as a response to community requests to develop a dataset of urban agriculture in Philadelphia. The GDC consists of several universities, community members, and grassroots organizations including Public Interest Law Center of Philadelphia, Soil Generation, Pennsylvania Horticultural Society, Neighborhood Garden Trust, Haverford College, and Villanova.

The dependent variable of interest is the count of community gardens and urban farms within a block group. Community gardens and urban farms were spatially joined to the block group in which they are located based on its centroid. For the remainder of this paper, we refer to community gardens and farms interchangeably with “urban agriculture” and “community food-growing spaces.”

The dataset builds on data collected and cleaned by Vitiello and Nairn for the 2008 Harvest Report: Community Gardening in Philadelphia (Vitiello and Nairn, 2008). Original methods are outlined there. Starting with this initial dataset, the GDC began adding data from multiple sources including the Pennsylvania Horticultural Society, who had data on all new community gardens in Philadelphia after 2008. From there, additional geocoding, data cleaning, and verifications were completed by a team of researchers and community gardeners from several Philly-area universities, foundations, and organizations (Bouffard, 2017). Verification included physically visiting suspected garden sites and using satellite imagery to confirm their existence. After cleaning and confirming garden locations, the GDC joined gardens with parcel data from the Public Works department. Cleaning, coding, and confirmation of the new data spanned several years from 2014 to 2017.

In 2019, the dataset was transferred to one of the City of Philadelphia’s project teams to begin a secondary process of verifying garden locations to determine if the included sites were still active, demolished, and/or developed. Through a rigorous community engagement process, this project team gathered data on new community garden and urban farm locations through in-person public meetings, virtual interviews, and online survey forms. Additional sites were confirmed through in-person visits, satellite imaging, and interviews with community leaders. While satellite imaging was used in this project to encourage the expansion of community-led urban agriculture in Philadelphia, it does have potential to be used for predatory land practices. As researchers using the latest technology, we must ensure our work does not harm the communities or people that we have a responsibility to protect.

### 3.3. Statistical analysis

We calculated descriptive statistics for the block groups included in this study. We created maps depicting spatial distribution of quintiles of percent Black, poverty, and food access within block groups. We also produced maps where community garden and farm locations were plotted alongside block group demographics. All maps were created using R.

We used several regression models to assess the associations between block group neighborhood characteristics and urban agriculture in Philadelphia. For each primary independent variable, we separately conducted three sets of analyses: unadjusted Poisson regression, Poisson regression adjusted for covariates, and spatial Poisson regression adjusted for covariates. We suspected possible residual spatial auto-correlation based on Tobler’s Law – that block groups closer together are more likely to be similar than block groups further apart – and used a Besag York Mollie (BYM) spatial random effects Poisson model (Besag et al., 1991) to account for it. An offset term for log of population size was included to model the count of community gardens and urban farms as a rate per person.

We conducted spatial sensitivity analyses to determine if results differed significantly with a dichotomized outcome. The dichotomized outcome was 1 – if a block group had at least one community garden or urban farm, and 0 – if a block group had no community gardens or farms. We also ran an analysis to determine if the association between low food access and the presence of community gardens differed based on the percentage of a block group identifying as Black.

## 4. Results

**Fig. 1.**
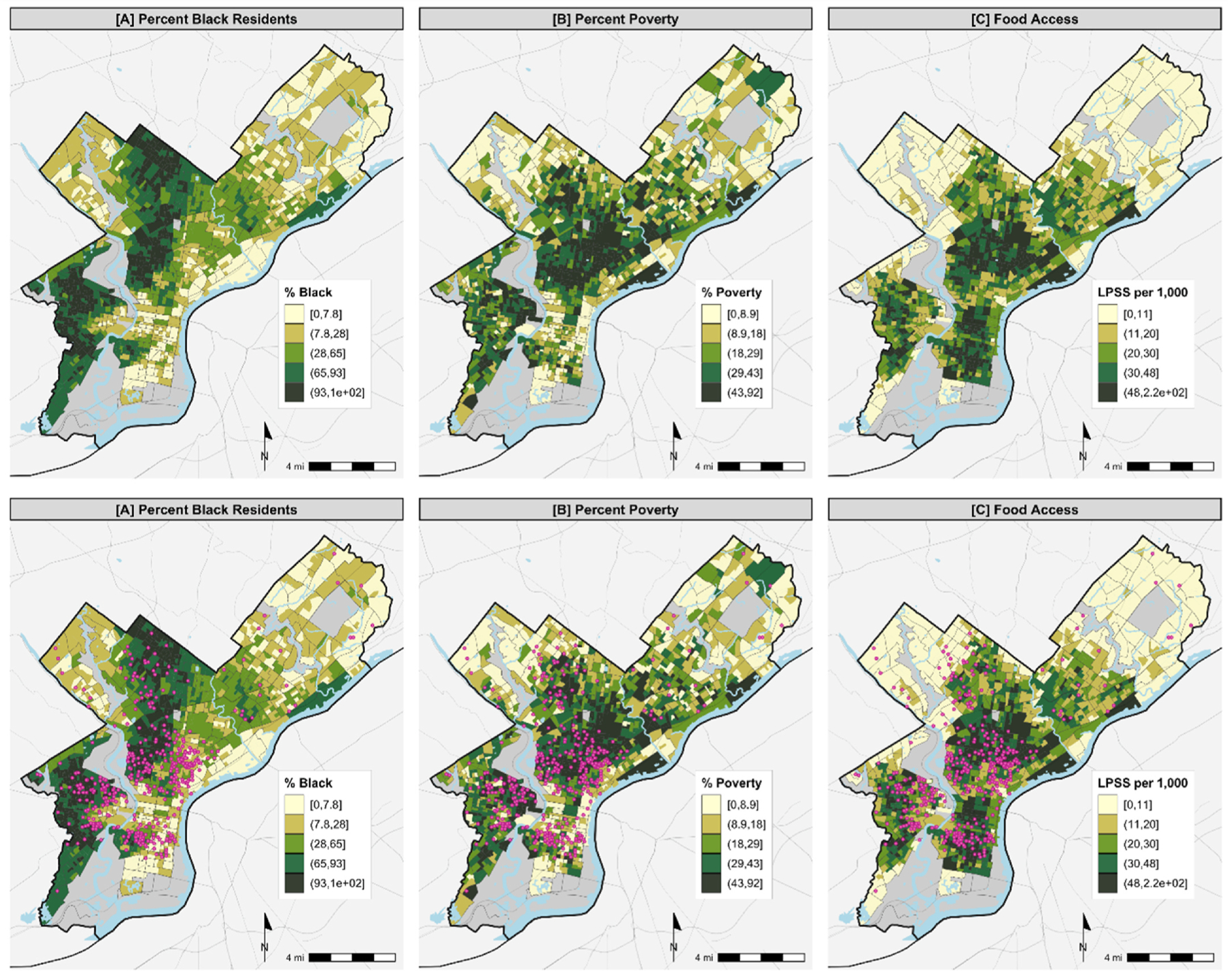
Maps of Independent Variables and Community Food-Growing Spaces in Philadelphia. Pink dots represent the location of a community garden or urban farm.. (For interpretation of the references to color in this figure legend, the reader is referred to the Web version of this article.)

Table 1 and Fig. 1 show descriptive statistics and maps of percent Black, poverty, and low food access by block group. The average percentage of Black people in a block group was 49%, with an average of 27% of people in a block group living in poverty. About 21% of block groups contained one or more community gardens or urban farms (n = 283).

Univariable analyses show significant associations of Black neighborhoods (p < 0.001), poverty (p < 0.001), and low food access (0.001) with urban agriculture (Table 2). Fig. 1 highlights the location of community gardens and urban farms in relation to neighborhood demographics.

Fully adjusted spatial models (Table 2) showed significant associations between Black neighborhoods and urban agriculture (RR: 1.28, 95% CI = 1.03, 1.59) and poverty and urban agriculture (RR: 1.27, 95% CI = 1.1, 1.46). Low food access and urban agriculture were positively associated, but the confidence interval contains the null value (95% CI = —0.031, 0.256). Block groups with a 1 standard deviation increase in the percentage of Black people had a 28% increase in rate of community gardens. Similarly, block groups with a 1 standard deviation increase in percent poverty had a 27% increase in rate of community gardens.

**Fig. 2.**
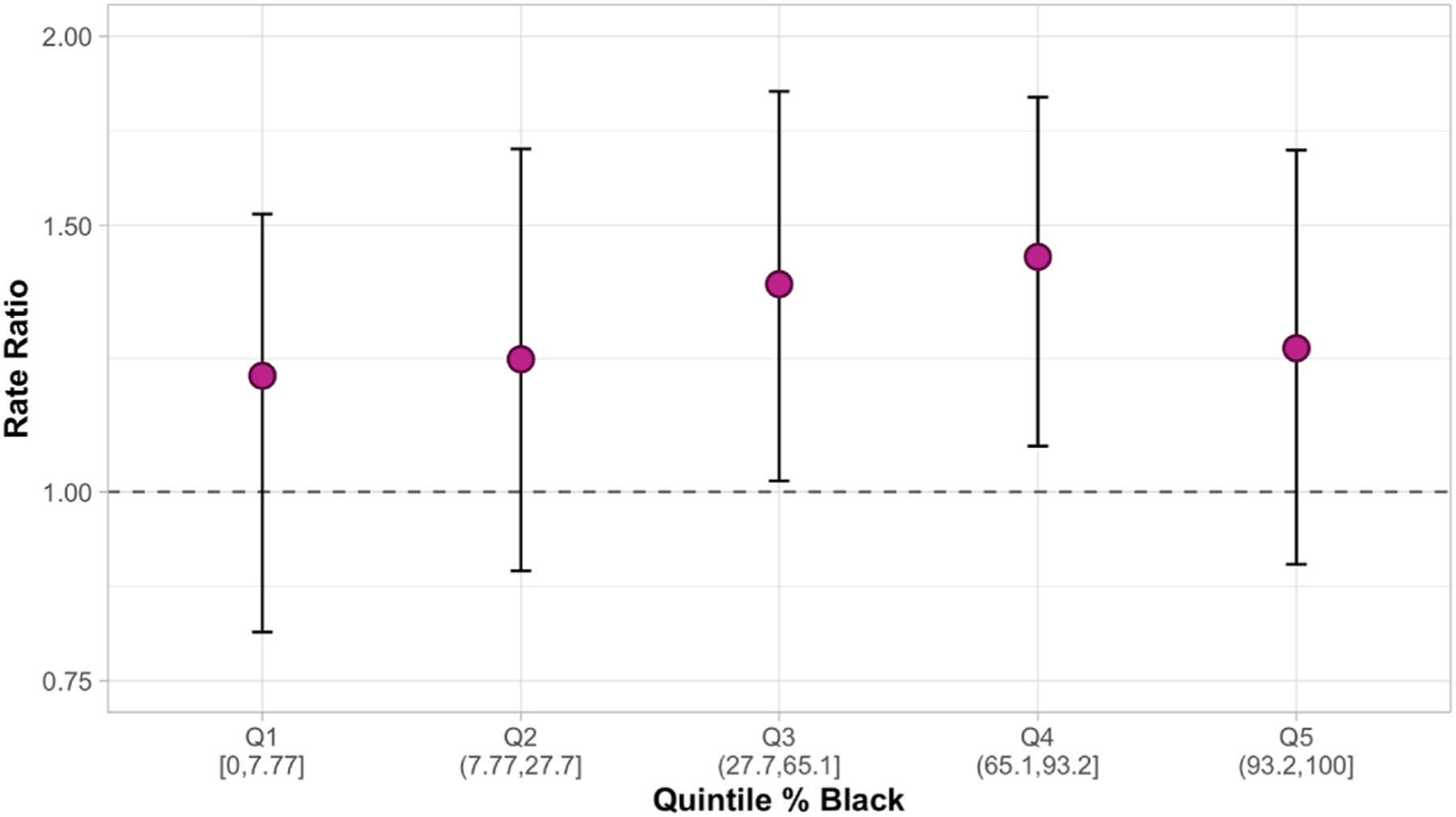
Rate Ratio and 95% confidence interval (CI) for the change in rate of community gardens (outcome) for every one standard deviation (SD) increase in low food access (exposure) by neighborhood quintile of percent Black residents (modifier). Rate ratio and 95% CIs estimated from spatial models with an interaction term between the continuous low food access variable and the categorical variable, quintile of percentage Black residents.

Black neighborhoods, poverty, and food access were all associated with the dichotomized urban agriculture variable. Another sensitivity analysis including all independent variables in the same model yielded similar results – Black neighborhoods and poverty were each significantly associated with urban agriculture while food access was not. Fig. 2 looks at whether neighborhoods that have high concentrations of Black people and low food access were more likely to have urban agriculture presence. After stratifying neighborhoods into quintiles by percentage of Black residents, we found that the association between low food access and community urban agriculture generally increased across quintiles of neighborhoods with a higher percentage of Black residents. What this means is, the association between low food access and community gardens was stronger in Black neighborhoods. Although this analysis is not causal and non-temporal, it does align with what community members have said: that as Black neighborhoods continue to experience neglect and food apartheid, community members respond by establishing healing gardens and food growing spaces.

## 5. Discussion

The goal of this work was to explore the landscape of Philadelphia’s community food-growing spaces in the context of neighborhood-level variables such as racial composition, food access, and income.

We chose to look at each of our primary independent variables separately because we believe racial composition, poverty, and food access each tell us something important about the manifestation of structural racism as environmental racism. Due to centuries of racist policies and practices, Black neighborhoods tend to experience food apartheid (Alkon and Agyeman, 2011; Mishan, 2021) resulting in higher concentrations of poverty and less access to nutritious foods (Jeffery and French, 1998; Block et al., 2004; Berger et al., 2019; Zenk et al., 2005; Reidpath et al., 2002; Morland et al., 2006). As such, each of our independent variables – racial composition, poverty, and food access – are interrelated manifestations of environmental racism and do no operate independently. Our goal is to understand the relationships between neighborhood racial composition, poverty, and low food access, not to control for or explain away their statistical effects on each other (Kawachi et al., 2005). Instead, we believe it’s important to look at them all as distinct, mutually reinforcing social constructs that, together, contribute to the presence of urban agriculture in Black neighborhoods.

Our results show that Philadelphia neighborhoods with higher populations of Black people and neighborhoods with higher concentrations of poverty, on average, have more community gardens and urban farms. While the garden data is non-temporal and these analyses are non-causal, one possible explanation for these findings is that urban agriculture is a manifestation of collective agency and community resistance in Black neighborhoods. Growers in Philly have said that establishing gardens and farms has historically been a way for communities to feed themselves, care for themselves, and heal themselves in the face of extraction (Strauss, 2019).

Gardens are a potential self-determined path to community healing and wellbeing. Since gardens tend to be concentrated in Black neighborhoods, poor neighborhoods, and areas with low food access, it is not unreasonable to suggest that community gardens and urban farms may be a response to food apartheid and structural oppression in Philadelphia. Future studies should not only look at who is growing, what they are growing and where, but also examine *why* people are growing to better understand possible mechanisms through which Black-led agriculture impacts the health of communities and individuals.

This study was somewhat limited by the non-temporal nature of the dataset which makes it more difficult to say if the racial composition and poverty status of block groups precedes the presence of urban agriculture. We note, however, that the reverse scenario is not entirely plausible. Green space, particularly well-kept green space, raises the property values of neighborhoods. It is unlikely that the presence of community gardens would lead to block groups having higher percentages of Black people and/or people living in poverty. In general, structural and environmental racism manifest in a way that tries to limit Black people’s access to well-kept and cultivated green space (Bailey et al., 2021).

Another limitation of this study lies within the Philadelphia Neighborhood Food Retail dataset. There is no clear measure or consideration given to the quality of stores, other than being defined as having a high produce supply or low produce supply. To adjust for this, we opted to use the number of low produce supply stores as an indicator of food access. This measure seems to capture access, or lack thereof, to nutritious, high-quality produce more adequately.

Lastly, the data on community gardens and urban farms has some missingness with respect to the type of garden. For instance, the categories include individual gardens, community gardens, school gardens, prison gardens, urban farms, market farms, and community spaces. The category with the most missingness and uncertainty is “individual gardens.” While it is concerning that there is a moderate degree of missingness in this category, it is neither surprising nor does it impact this analysis as we are looking only at community gardens and urban farms.

Our findings show that Black neighborhoods and low-income neighborhoods in Philadelphia tend to have higher concentrations of community food-growing spaces. These results strengthen the case to increase access to nourishing, nutritious, affordable, and locally grown food for Black people and low-income communities. Increasing access to and participation in urban agriculture for Black people may also help to reduce stress, anxiety, and depression, while also increasing collective agency and social support within these communities. Beyond its impact on physical health, urban agriculture is thought to be a potential strategy to help cities become more self-reliant by utilizing sustainable food sources (Grewal and Grewal, 2012).

Public Health and Policy Implications: Land Security and Philadelphia First Urban Agriculture Plan.

Philly community gardens and urban farms may also attract developers who are interested in profiting off the time, energy, and resources that Black and immigrant communities have dedicated to creating healing growing spaces (Strauss, 2019; Crouch, 2012; Melamed, 2021; Wozniacka, 2019; Gervasi, 2019). Well-kept green space in Philadelphia raises the property values in a neighborhood. As the monetary value of these transformed lots increases, developers and city officials have sometimes threatened and followed through with bulldozing community-growing spaces to make space for new development (Crouch, 2012; Melamed, 2021; Wozniacka, 2019; Gervasi, 2019). This has occurred in Philadelphia through sheriff sales and city-council sales of vacant land directly to developers (Bender, 2016).

Currently, 1 in 3 of Philly’s active gardens or farms are in areas with the greatest intensity of new construction (DiStasi et al., 2021). Over 56% of gardens and farms do not have land security (DiStasi et al., 2021). This means that these growing spaces are located on land that is not owned by the people caring for them, and they are at risk of being sold to developers. Of these, about 74% are currently on city-owned land allowing for a potential pathway to true land security. City agencies and officials such as the Philadelphia Land Bank and City Council could impact healthy food access and strengthen social capital and collective agency in neighborhoods if they adopt and implement policies that support urban agriculture instead of policies that are roadblocks for growers seeking land security.

Philadelphia is currently developing its first Urban Agriculture Plan and the project team hopes to do so in a way that considers, contextualizes, and uplifts racial justice, economic justice, climate justice, community building, culture, history, and healing (Jaramillo, 2021; City of Philadelphia Kicks Off, 2021). The plan’s goal is to support all Philadelphians with emphasis on supporting the livelihoods and work of community members who grow food and are most impacted by food apartheid. This study’s findings echo that the neighborhoods with the greatest urban agriculture presence and economic insecurity are populated with the very people that the urban agriculture plan hopes to most support. One way to support the preservation and expansion of urban gardens is for city council and landholding agencies to implement the recommendations put forth in Growing from the Root: Philadelphia’s Urban Agriculture Plan.

Many gardens, farms, and community-growing spaces are currently located on tax-delinquent and foreclosed lots. This is because these lots tend to be vacant and abandoned. The lots that house these gardens and farms often go up for sheriff sale without notification to the people currently caring for them. Sheriff sales have allowed many properties and lots in Philadelphia to be purchased by real estate investors and developers who do not live in the communities in which they are purchasing land, nor are they invested in the livelihoods of the people who live in those neighborhoods.

This is just one example of a modern strategy for acquiring land that has been stewarded and cared for by Black and immigrant growers in Philadelphia. Despite these examples of inequitable policies and food apartheid, Philadelphia’s food and agriculture presence should be characterized by the Black and immigrant communities who exercise collective self-determination, agency, and resistance.

Our results show concentrations of low produce supply stores in Black and low-income neighborhoods throughout the city. We hope to see Philadelphia policymakers implement policies and practices that support community gardens, urban farms, and farmer’s markets. As mentioned in Mayor Jim Kenney’s report “Equity and Opportunity for all,” (D’Onofrio, 2020) the City of Philadelphia claims to care about improving the health of its communities. This means the City also needs to prioritize agriculture because of the deep connections that growing food has to the health of Philly’s communities.

Increased access to fresh, nutritious, and locally grown foods in low-income neighborhoods can have benefits that extend beyond health. These benefits include increased community building and healing, a strengthened local economy, and community job development (Golden, 2013; Kaufman and Bailkey, 2000; FAO’s role in Urban Agriculture, 2019; Santo et al., 2016). Black and low-income neighborhoods are the exact areas where urban agriculture has had and can continue to have the greatest impacts.

## Figures and Tables

**Table 1:** Descriptive statistics for block groups in Philadelphia (n = 1324).

Variable	Mean (SD)
Percent *(Black)*	48.81 (37.29)
Percent Poverty	26.56 (18.83)
Low Produce Supply Stores (per 1000 people)	30.95 (25.27)
Median House Value^a^	230.38 (143.97)
Population Density^a^	23,354 (14,475)
**Frequency (%)**	
Community Gardens and Urban Farms	
0 gardens or farms	1041 (78.62)
1 garden and farms	196 (14.80)
2 gardens and farms	57 (4.31)
3 or more gardens and farms	30 (2.27)

a Median house value is in units of $100,000. Population density refers to the number of people in a block group.

**Table 2:** Univariable and multivariable rate ratio spatial models for primary independent variables including covariates. These models include an offset term for population to model count as rate.

	Univariable RR (SE)	p-value	Multivariable Spatial RR	Confidence Interval
**Race (Black)**	1.34 (0.049)	(p < 0.001)	1.28	1.026, 1.59
**Poverty**	1.50 (0.043)	(p < 0.001)	1.27	1.10, 1.46
**Low Food Access (Low Produce Supply Stores)**	1.48 (0.037)	(p < 0.001)	1.13	0.97, 1.29

## Positionality

Being active in food justice and land sovereignty movements for over decade have allowed me to establish connections and build meaningful relationships with urban and rural growers from around the country. I was born and raised in Philadelphia where my family has been for five generations. I am a member of Soil Generation, a Black and brown coalition of farmers, gardeners, and food justice activists in Philadelphia. I am also the founder of a small organization that provides education to Black women on agriculture, building, safety, and land-based living. My position allows me to access intimate Black agriculture spaces and lends perspective to the methods and questions I engage in my research. While my involvement in this community helps me to articulate more impactful research questions and approaches, it does not impact the results and findings of my scholarship.

## Appendix

**Table 3:** Conditional Autoregression BYM including all independent variables and covariates in a single model.

	RR	2.5%	97.5%
**Race (Black)**	1.26	1.004	1.55
**Poverty**	1.25	1.056	1.43
**Food Access (Low)**	1.15	0.996	1.32
House Value	0.901	0.764	1.04
Population Density	0.694	0.694	0.694

**Table 4:** Sensitivity analysis – binary outcome of community gardens and urban farms including covariates.

	Estimate	p-value	Standard error
**Race (Black)**	1.16	<2e-16	0.071
**Poverty**	1.51	1.38–08	0.073
**Food Access (Low)**	1.19	0.0003	0.077

## References

[R1] Alaimo K, Beavers AW, Crawford C, Snyder EH, Litt JS, 2016. Amplifying health through community gardens: a framework for advancing multicomponent, behaviorally based neighborhood interventions. Curr Environ Health Rep 3 (3), 302–312. 10.1007/s40572-016-0105-0. 27379424

[R2] Alkon AH, Agyeman J, 2011. Cultivating Food Justice: Race, Class, and Sustainability. The MIT Press. 10.7551/mitpress/8922.001.0001.

[R3] Audate PP, Fernandez MA, Cloutier G, Lebel A, 2019. Scoping review of the impacts of urban agriculture on the determinants of health. BMC Publ. Health 19. 10.1186/s12889-019-6885-z.

[R4] Bailey ZD, Krieger N, Agénor M, Graves J, Linos N, Bassett MT, 2017. Structural racism and health inequities in the USA: evidence and interventions. Lancet Br Ed 389 (10077), 1453–1463. 10.1016/S0140-6736(17)30569-X. 28402827

[R5] Bailey ZD, Feldman JM, Bassett MT, 2021. How structural racism works — racist policies as a root cause of U.S. Racial health inequities. In: Malina D (Ed.), N. Engl. J. Med 384 (8), 768–773. 10.1056/NEJMms2025396. 33326717 PMC11393777

[R6] Bender W, August 7, 2016. Developers bought low and sold high with the help of Philadelphia city councilman kenyatta johnson. Phila. Inq. (PA) https://www.inquirer.com/news/inq/developers-bought-low-sold-high-with-help-philadelphia-city-councilman-kenyatta-johnson-20160806.html. (Accessed 27 September 2021).

[R7] Berger N, Kaufman TK, Bader MDM, , 2019. Disparities in trajectories of changes in the unhealthy food environment in New York City: a latent class growth analysis, 1990–2010. Soc. Sci. Med 234, 112362 10.1016/j.socscimed.2019.112362. 31247345 PMC6689383

[R8] Besag J, York J, Molli A, 1991. Bayesian image restoration, with two applications in spatial statistics. Ann. Inst. Stat. Math 43 (1), 1–20. 10.1007/BF00116466.

[R9] Birch E, Wachter S, 2008. Growing Greener Cities: Urban Sustainability in the Twenty-First Century. University of Pennsylvania Press. http://muse.jhu.edu/book/12014. (Accessed 23 September 2021).

[R10] Block JP, Scribner RA, DeSalvo KB, 2004. Fast food, race/ethnicity, and income: a geographic analysis. Am. J. Prev. Med 27 (3), 211–217. 10.1016/j.amepre.2004.06.007. 15450633

[R11] Borowiak C, Safri M, Healy S, Pavlovskaya M, 2018. Navigating the fault lines: race and class in Philadelphia’s solidarity economy. Antipode 50 (3), 577–603. 10.1111/anti.12368.

[R12] Bouffard M, 2017. How a local data collaborative increases value of community gardens. Green Philly Published March 23. https://www.thegreencities.com/philly/the-city-of-brotherly-love-and-urban-gardens/. (Accessed 23 September 2021).

[R13] Bradley K, Galt RE, 2014. Practicing food justice at Dig Deep Farms & Produce, East Bay Area, California: self-determination as a guiding value and intersections with foodie logics. Local Environ 19 (2), 172–186. 10.1080/13549839.2013.790350.

[R14] Briggs R Philly is finally selling its vacant lots. Now the question is who will benefit. Plan Philly Accessed September 23, 2021. https://whyy.org/articles/philadelphia-land-bank-is-finally-selling-its-vacant-lots-now-the-question-is-who-will-benefit/.

[R15] Brones A, May 15, 2018. Food Apartheid: the Root of the Problem with America’s Groceries. The Guardian. http://www.theguardian.com/society/2018/may/15/food-apartheid-food-deserts-racism-inequality-america-karen-washington-interview. (Accessed 3 August 2021).

[R16] Bullard RD, 1993. The threat of environmental racism. Nat. Resour. Environ 7 (3), 23–56.

[R17] Burt KG, Mayer G, Paul R, 2021. A systematic, mixed studies review of the outcomes of community garden participation related to food justice. Local Environ 26 (1), 17–42. 10.1080/13549839.2020.1861589.

[R18] Philadelphia’s Changing Neighborhoods, 2016. Gentrification and Other Shifts since 2000. The PEW Charitable Trusts. http://pew.org/1TXTXY7. (Accessed 29 April 2022).

[R19] City of Philadelphia kicks off first urban agriculture planning process | Philadelphia parks & recreation. City of Philadelphia. In: https://www.phila.gov/2019-10-22-city-of-philadelphia-kicks-off-first-urban-agriculture-planning-process/. (Accessed 27 September 2021).

[R20] Crouch P Evolution or gentrification: do urban farms lead to higher rents? Grist. Published online October 23, 2012. Accessed September 27, 2021. https://grist.org/food/evolution-or-gentrification-do-urban-farms-lead-to-higher-rents/.

[R21] Daniel P, 2013. Dispossession: Discrimination against African American Farmers in the Age of Civil Rights. University of North Carolina Press. www.jstor.org/stable/10.5149/9781469602028_daniel. (Accessed 23 November 2019).

[R22] DiStasi C, Gripper A, Galiber S, Baxter K, Gonzalez M, Watts M, 2021. Station 3: how can we get access to land? Growing from the root: Philadelphia’s urban agriculture plan. https://sites.google.com/interface-studio.com/publicmeeting2/stations/station-3. (Accessed 23 September 2021).

[R23] Douglas L African Americans have lost untold acres of land over the last century. Published online June 26, 2017. https://www.thenation.com/article/african-americans-have-lost-acres/. (Accessed 23 November 2019).

[R24] Draper C, Freedmand D, 2010. Review and analysis of the benefits, purposes, and motivations associated with community gardening in the United States: journal of community practice: vol 18, No 4. J. Community Pract 18 10.1080/10705422.2010.519682.

[R25] D’Onofrio M, January 6, 2020. Kenney signs orders on racial equity, reshaping Office of Education as he lays out 2nd-term priorities. Phila. Tribune Published. https://www.phillytrib.com/news/local_news/kenney-signs-orders-on-racial-equity-reshaping-office-of-education-as-he-lays-out-2nd/article_a73236b5-0083-5569-a12b-e12797ec0a87.html. (Accessed 27 September 2021).

[R26] Fang D, Thomsen MR, Nayga RM, 2021. The association between food insecurity and mental health during the COVID-19 pandemic. BMC Publ. Health 21 (1), 607. 10.1186/s12889-021-10631-0. PMC8006138 33781232

[R27] FAO’s role in urban agriculture. Food and agriculture organization of the united nations. http://www.fao.org/urban-agriculture/en/. (Accessed 23 November 2019).

[R28] Ficara JF, Williams J, 2005. Black farmers in America.” NPR.org. https://www.npr.org/2005/02/22/5228987/black-farmers-in-america. (Accessed 23 November 2019).

[R29] Garcia MT, Ribeiro SM, Germani ACCG, Bógus CM, 2018. The impact of urban gardens on adequate and healthy food: a systematic review. Publ. Health Nutr 21 (2), 416–425. 10.1017/S1368980017002944. PMC10260856 29160186

[R30] George DR, 2013. Harvesting the biopsychosocial benefits of community gardens. Am. J. Publ. Health 103 (8). 10.2105/AJPH.2013.301435 e6-e6. PMC4007866 23763423

[R31] Gervasi A At Philly’s community gardens, a growing frustration over their future. WHYY Plan Philly Published online May 29, 2019. Accessed September 27, 2021. https://whyy.org/articles/at-phillys-community-gardens-growing-frustration-over-their-future/.

[R32] Girard AW, Self JL, McAuliffe C, Olude O, 2012. The effects of household food production strategies on the health and nutrition outcomes of women and young children: a systematic review. Paediatr. Perinat. Epidemiol 26 (s1), 205–222. 10.1111/j.1365-3016.2012.01282.x. 22742612

[R33] Golden S, 2013. Urban Agriculture Impacts: Social, Health, and Economic: an Annotated Bibliography. University of California, p. 22.

[R34] Great migration. HISTORY. https://www.history.com/topics/black-history/great-migration. (Accessed 24 November 2019).

[R35] Gregis A, Ghisalberti C, Sciascia S, Sottile F, Peano C, 2021. Community garden initiatives addressing health and well-being outcomes: a systematic review of infodemiology aspects, outcomes, and target populations. Int. J. Environ. Res. Publ. Health 18 (4), 1943. 10.3390/ijerph18041943. PMC7922762 33671320

[R36] Grewal SS, Grewal PS, 2012. Can cities become self-reliant in food? Cities 29 (1), 1–11. 10.1016/j.cities.2011.06.003.

[R37] Gripper A We don’t farm because it’s trendy; we farm as resistance, for healing and sovereignty. Agents of Change in Environmental Health. Published May 27, 2020. https://www.ehn.org/black-farming-food-sovereignty-2645479216.html. (Accessed 3 August 2021).

[R38] Gyourko J, Margo RA, Haughwout AF, 2005. Looking back to look forward: learning from Philadelphia’s 350 Years of urban development [with comments]. Brook-Whart Pap Urban Aff 1–58. Published online.

[R39] Here’s the dirt on park trends: Community gardens are growing. The Trust for Public Land. https://www.tpl.org/blog/here%E2%80%99s-dirt-park-trends-community-gardens-are-growing. (Accessed 23 September 2021).

[R40] Jaramillo C Philly’s first urban agriculture director kicks off plan to save endangered edible gardens. WHYY Plan Philly Accessed September 27, 2021. https://whyy.org/articles/phillys-farm-chief-kicks-off-plan-to-save-endangered-edible-gardens-and-create-more-on-vacant-city-lots/.

[R41] Jeffery RW, French SA, 1998. Epidemic obesity in the United States: are fast foods and television viewing contributing? Am. J. Publ. Health 88 (2), 277–280. 10.2105/ajph.88.2.277. PMC1508201 9491022

[R42] Kaufman J, Bailkey M, 2000. Farming inside Cities: Entrepreneurial Urban Agriculture in the United States, p. 124.

[R43] Kawachi I, Daniels N, Robinson DE, 2005. Health disparities by race and class: why both matter. Health Aff 24 (2), 343–352. 10.1377/hlthaff.24.2.343. 15757918

[R44] Kondo MC, Fluehr JM, McKeon T, Branas CC, 2018. Urban green space and its impact on human health. Int. J. Environ. Res. Publ. Health 15 (3), 445. 10.3390/ijerph15030445. PMC5876990 29510520

[R45] Lampert T, Costa J, Santos O, Sousa J, Ribeiro T, Freire E, 2021. Evidence on the contribution of community gardens to promote physical and mental health and well-being of non-institutionalized individuals: a systematic review. PLoS One 16 (8), e0255621. 10.1371/journal.pone.0255621. 34358279 PMC8345884

[R46] Larson NI, Story MT, Nelson MC, 2009. Neighborhood environments: disparities in access to healthy foods in the U.S. Am. J. Prev. Med 36 (1), 74–81. 10.1016/j.amepre.2008.09.025. 18977112

[R47] Laskawy T, 2010. Philadelphia’s urban-farming roots go deep – and are spreading wide. Grist Published online September 22. https://grist.org/article/food-2010-09-21-philadelphias-urban-farming-roots-go-deep-and-are-spreading/. (Accessed 23 September 2021).

[R48] Litt JS, Soobader MJ, Turbin MS, Hale JW, Buchenau M, Marshall JA. The Influence of Social Involvement, Neighborhood Aesthetics, and Community Garden Participation on Fruit and Vegetable Consumption. 10.2105/AJPH2010300111. Published online October 20, 2011. doi:10.2105/AJPH2010300111. PMC3134498 21680931

[R49] Manson Steven, Schroeder Jonathan, Riper Van, David Kugler, Tracy Ruggles, Steven. National Historical Geographic Information System: Version 16.0. Published online 2021. doi:10.18128/D050.V16.0.

[R50] McClintock N, 2014. Radical, reformist, and garden-variety neoliberal: coming to terms with urban agriculture’s contradictions. Local Environ 19 (2), 147–171. 10.1080/13549839.2012.752797.

[R51] McCormack LA, Laska MN, Larson NI, Story M, 2010. Review of the nutritional Implications of farmers’ markets and community gardens: a call for evaluation and research efforts. J. Am. Diet Assoc 110 (3), 399–408. 10.1016/j.jada.2009.11.023. 20184990

[R52] Melamed S Philly’s urban gardeners are under siege from gentrification. Here’s what they’re doing about it. https://www.inquirer.com. (Accessed 27 September 2021). https://www.inquirer.com/philly/news/pennsylvania/philadelphia/urban-garden-philly-farm-phs-pennsylvania-horticultural-society-ngt-neighborhood-gardens-trust-20180416.html.

[R53] Mishan L, February 19, 2021. The activists working to remake the food system. N. Y. Times. September 27, 2021. https://www.nytimes.com/2021/02/19/t-magazine/food-security-activists.html.

[R54] Morland K, Diez Roux AV, Wing S, 2006. Supermarkets, other food stores, and obesity: the atherosclerosis risk in communities study. Am. J. Prev. Med 30 (4), 333–339. 10.1016/j.amepre.2005.11.003. 16530621

[R55] Nairn M, 2007. Keeping the community in community gardening: aqui estamos y no nos vamos – planners network. PN Plan Netw Published online. https://www.plannersnetwork.org/2007/01/keeping-the-community-in-community-gardening-aqui-estamos-y-no-nos-vamos/. (Accessed 23 September 2021).

[R56] Nairn M, Vitiello D, 2009. Lush lots: everyday urban agriculture from community gardening to community food security. Harv. Des. Mag 31, 94–161.

[R57] Neighborhood Food Retail. Food fit philly http://foodfitphilly.org/neighborhoodfoodretail/. (Accessed 23 September 2021).

[R58] Odoms-Young A, Bruce MA, 2018. Examining the impact of structural racism on food insecurity Implications for addressing racial/ethnic disparities. Fam. Community Health 41 (2), S3–S6. 10.1097/FCH.0000000000000183. 29461310 PMC5823283

[R59] Ohly H, Gentry S, Wigglesworth R, Bethel A, Lovell R, Garside R, 2016. A systematic review of the health and well-being impacts of school gardening: synthesis of quantitative and qualitative evidence. BMC Publ. Health 16, 286. 10.1186/s12889-016-2941-0. PMC4807565 27015672

[R60] One million black families in the South have lost their farms over the past century. Equal justice initiative. https://eji.org/news/one-million-black-families-have-lost-their-farms/, October 11, 2019–. (Accessed 24 November 2019).

[R61] Park IK, Ciorici P, 2013. Determinants of vacant lot conversion into community gardens: evidence from Philadelphia. Int J Urban Sci 17 (3), 385–398. 10.1080/12265934.2013.818388.

[R62] Reidpath DD, Burns C, Garrard J, Mahoney M, Townsend M, 2002. An ecological study of the relationship between social and environmental determinants of obesity. Health Place 8 (2), 141–145. 10.1016/s1353-8292(01)00028-4. 11943585

[R63] Rosenberg N, Stucki BW. How USDA distorted data to conceal decades of discrimination against black farmers. New Food Economy. https://newfoodeconomy.org/usda-black-farmers-discrimination-tom-vilsack-reparations-civil-rights/. Published June 26, 2019. Accessed November 23, 2019.

[R64] Rothstein R, 2017. The Color of Law: A Forgotten History of How Our Government Segregated America, first ed. Liveright Publishing Corporation, a division of WWNorton & Company.

[R65] Santo R, Palmer A, Kim B, 2016. Vacant Lots to Vibrant Plots: A Review of the Benefits and Limitations of Urban Agriculture. Johns Hopkins University. https://clf.jhsph.edu/. (Accessed 23 November 2019).

[R66] Scranton P, 1992. Large firms and industrial restructuring: the Philadelphia region, 1900–1980. Pa. Mag. Hist. Biogr 116 (4), 419–465.

[R67] Speirs FW, 1898. Vacant-lot cultivation. New York, charities review. http://archive.org/details/vacantlotcultiva00speiuoft. (Accessed 23 September 2021).

[R68] Strauss M, 2019. Back to the land: struggles over food and real estate development in Philadelphia. New Labor Forum 28 (2), 79–83. 10.1177/1095796019836747.

[R69] Tatah M, 2017. Africa in the Colonial Ages of Empire: Slavery, Capitalism, Racism, Colonialism, Decolonization, Independence as Recolonization, and beyond. Langaa RPCIG

[R70] The top 10 leading causes of death in the United States. Medical News Today. https://www.medicalnewstoday.com/articles/282929.php. (Accessed 24 November 2019).

[R71] Tolnay SE, 2003. The African American “great migration” and beyond. Annu Rev Sociol Palo Alto 29, 209–232. 10.1146/annurev.soc.29.010202.100009.

[R72] Tyler S, Moore E, 2013. Plight of black farmers in the context of USDA farm loan programs: a research agenda for the future. Prof Agric Work J 1 (1). https://tuspubs.tuskegee.edu/pawj/vol1/iss1/6.

[R73] Vacant Lot Program | Community Life Improvement Program. City of Philadelphia. https://www.phila.gov/programs/vacant-lot-program/. (Accessed 3 August 2021).

[R74] Vitiello D, Nairn M Community gardening in Philadelphia 2008 harvest report. Penn planning and urban studies. University of Pennsylvania. https://www.millcreekurbanfarm.org/sites/default/files/Philadelphia%20Harvest%20%28with%20images%29.pdf, 68.

[R75] White MM, 2010. Shouldering responsibility for the delivery of human rights: a case study of the D-town farmers of Detroit. RaceEthnicity Multidiscip Glob Contexts 3 (2), 189–211.

[R76] White MM, 2011. Environmental reviews & case studies: D-town farm: african American resistance to food insecurity and the transformation of Detroit. Environ. Pract 13 (4), 406–417. 10.1017/S1466046611000408.

[R77] White MM, 2018. Freedom Farmers: Agricultural Resistance and the Black Freedom Movement. UNC Press Books.

[R78] Wozniacka G Soil generation is saving community gardens in Philadelphia. Civ Eats Published online July 23, 2019. Accessed September 27, 2021. https://civileats.com/2019/07/23/soil-generation-is-saving-community-gardens-in-philadelphia/.

[R79] Zenk SN, Schulz AJ, Israel BA, James SA, Bao S, Wilson ML, 2005. Neighborhood racial composition, neighborhood poverty, and the spatial accessibility of supermarkets in metropolitan Detroit. Am. J. Publ. Health 95 (4), 660–667. 10.2105/AJPH.2004.042150. PMC1449238 15798127

